# Intergenerational Partnered Dance for PD and AD: Evaluating the Feasibility of the APPROACH Protocol

**DOI:** 10.1093/geroni/igaf122.4364

**Published:** 2025-12-31

**Authors:** Madeleine Hackney, Kozbi Bayne, Jill Bishop, Marcel Foster

**Affiliations:** Emory School of Medicine, Atlanta, Georgia, United States; Emory School of Medicine, Atlanta, Georgia, United States; Emory University School of Medicine, Atlanta, Georgia, United States; Performance Hypothesis, Atlanta, Georgia, United States

## Abstract

Dance, often conducted in an intergenerational context, may provide unique learning strategies that promote neuroplasticity to mitigate Parkinson’s Disease (PD). Dance’s capacity to influence creativity is understudied. The Adaptango Performance Process for Older Adult Community Rehabilitation (APPROACH) protocol explores community building between People with PD and young adults. Partnering young adults with older adults with PD may lead to an intergenerational increase in creativity and understanding between generations, increasing social support and decreasing depression. We studied the intergenerational interactions occurring during a three-month choreographic process, using adapted tango dance. We define the impact of the instructor/older adult/young adult co-experiences and reveal the relationship between creativity and psychosocial status in older adults and younger adults. Six dyads of People with PD (71+/-9 y) and young adults (age= 21+/-2 y) participated in a three-month choreographic workshop involving classes twice per week. At baseline, participants completed cognitive and neuropsychological tests of creativity, and self-reports measuring social support and quality of life. Participants were qualitatively interviewed during the tenth choreographic class and at study end provided qualitative feedback about the extent to which APPROACH classes impacted their well-being and attitudes toward intergenerational relationships.  All participants performed in the final performance. Initial analyses show correlations between creativity, social support, and depression in younger adults and older adults with PD. The intergenerational approach may be effective for delivering therapy. Further implementation of the APPROACH protocol could lead to increased creativity and social support for young adults and older adults with PD.

